# Fluoride-Promoted Esterification (FPE) Chemistry: A Robust Route to Bis-MPA Dendrons and Their Postfunctionalization

**DOI:** 10.3390/molecules21030366

**Published:** 2016-03-17

**Authors:** Patrik Stenström, Oliver C. J. Andrén, Michael Malkoch

**Affiliations:** Department of Fibre and Polymer Technology, KTH Royal Institute of Technology, 10044 Stockholm, Sweden; pstens@kth.se (P.S.); oandren@kth.se (O.C.J.A.)

**Keywords:** dendron, dendritic polymers, polyesters, bis-MPA, FPE chemistry, carbonyl diimidazole, biotin, CuAAC click chemistry

## Abstract

Bifunctional dendrons based on 2,2-bis(methylol)propionic acid (bis-MPA) are highly desirable scaffolds for biomedical applications. This is due to their flawless nature and large and exact number of functional groups as well as being biodegradable and biocompatible. Herein, we describe a facile divergent growth approach to their synthesis from monobenzylated tetraethylene glycol and post functionalization utilizing fluoride-promoted esterification (FPE) chemistry protocols. The scaffolds, presenting selectively deprotectable hydroxyls in the periphery and at the focal point, were isolated on a multigram scale with excellent purity up to the fourth generation dendron with a molecular weight of 2346 Da in seven reactions with a total yield of 50%. The third generation dendron was used as a model compound to demonstrate its functionalizability. Selective deprotection of the dendron’s focal point was achieved with an outstanding yield of 94%, and biotin as well as azido functionalities were introduced to its focal point and periphery, respectively, through FPE chemistry. Bulky disperse red dyes were clicked through CuAAC to the dendron’s azido groups, giving a biotinylated dendron with multivalent dyes with a molecular weight of 6252 Da in a total yield of 37% in five reactions with an average yield of 82% starting from the third generation focally and peripherally protected dendron. FPE chemistry proved to be a superb improvement over previous protocols towards bis-MPA dendrons as high purity and yields were obtained with less toxic solvents and greatly improved monomer utilization.

## 1. Introduction

Dendritic polymers based on 2,2-bis(methylol)propionic acid (bis-MPA) belong to one of the most established families of dendritic polymers and were introduced by Hult *et al*. in the 1990s [1,2]. These commercially available scaffolds are biodegradable and biocompatible [3], with long shelf-life and simple synthetic protocols and they fulfill all the necessary criteria for future technological breakthroughs in the field of biomedicine. They are definitely a viable alternative to the popular polyamidoamine (PAMAM) dendrimers [4,5], especially in light of recent reports detailing poor shelf-life [6] and high inherent toxicity [3,7,8]. The excellent features of monodisperse bis-MPA dendrimers and dendrons have led to their exploitation as precision polymers in an array of potential applications [9,10], for instance, as drug delivery carriers [11], self-assembled monolayers on gold nanoparticles [12], highly structured honeycomb films [13,14], functional hydrogels [15,16] and soft tissue-adhesives [17]. The exceptional structural perfection of bis-MPA dendrimers have resulted in state-of-the-art MALDI-MS calibrants, SpheriCal^®^, that outperform current peptide and protein based standards [18].

To ensure the flawless structures of dendrimers and dendrons, the chemistry has to be highly efficient and selective. Traditionally, perfect bis-MPA scaffolds have been synthesized through a divergent growth approach utilizing the anhydride coupling of acetonide- or benzylidene-protected bis-MPA to hydroxyl groups at the periphery of the dendritic framework [19,20]. The prerequisite of these layer-by-layer reactions include the use of sensitizing *N,N*′-dicyclohexylcarbodiimide (DCC), toxic 4-(dimethylamino)pyridine (4-DMAP) and pyridine, and is often performed in dichloromethane as a preferred solvent. Most recently, fluoride-promoted esterification (FPE) was proposed as a modular and sustainable reaction to polyesters [21]. As a proof-of-concept, bis-MPA dendrimers up to the sixth generation were synthesized capitalizing on *in situ* imidazolide-activation of acetonide protected bis-MPA using carbonyldiimidazole (CDI) as a reagent. It was found that imidazolide-activated monomers react to full conversion with alcohols in the presence of cesium fluoride (CsF) to form esters. Dendrimers of high yields and quantities (above 90% on a 50 g scale for all reactions) and purity were isolated through simple extractions and all the reactions were conducted using ethyl acetate (EtOAc) as an environmentally friendly solvent. The FPE chemistry was also found compatible with click chemistry allowing rapid and orthogonal growth of bis-MPA dendrimers that eliminated typical iterative activation steps [22,23].

Higher generation dendrons based on bis-MPA are considered as high-value scaffolds due to their large and exact number of reactive groups as well as their dual-functionalization capacity. Their further manipulation with desired cargos needs to be exploited with outmost care with minimum loss of the pre-synthesized dendrons. Consequently, this paper details the synthesis of a unique platform of highly desirable bis-MPA dendrons from generation one to four utilizing FPE chemistry for both the growth as well as postfunctionalization reactions. The dendrons were synthesized via divergent growth from tetraethylene glycol (TEG) as a core with extended features. This strategy enables robust peripheral and core functionalizations of the dendrons via FPE chemistry with a minimal loss of valuable dendrons. As a proof of concept, a third generation dendron was successfully converted to a biologically interesting material comprising a single biotin in the core and multiple disperse red dyes at the periphery.

## 2. Results and Discussion

To date, bis-MPA dendrons were synthesized utilizing anhydride activated bis-MPA monomers via divergent growth. Furthermore, core postfunctionalization of the dendrons typically relies on robust click chemistry protocols [24,25] including copper-catalyzed azide-alkyne cycloaddition (CuAAC) [26] and TEC chemistry as a mean to circumvent the modification of traditional bis-MPA dendrons with a single carboxylic group at the focal point, recognized by their constrained nature and challenging functionalization. Generally the carboxylic group undergoes activation prior to the attachment to a desired group. In this case, the coupling efficiency is typically jeopardized mainly due to the mentioned steric constraints that are strongly dictated by the size and generation of the dendrons. An example is the limited yields achieved during the convergent synthesis of bis-MPA dendrimers from higher generation dendrons. The covalent attachment via DCC activation of third generation dendrons, added in excess, to a triol core resulted in loss of 33% of the added dendrons [1]. Subsequently, by introducing an alcohol at the focal point being partly distanced from the framework, the functionalization of this position can be carried out with maximized efficiency of the valuable dendron.

**Figure 1:**
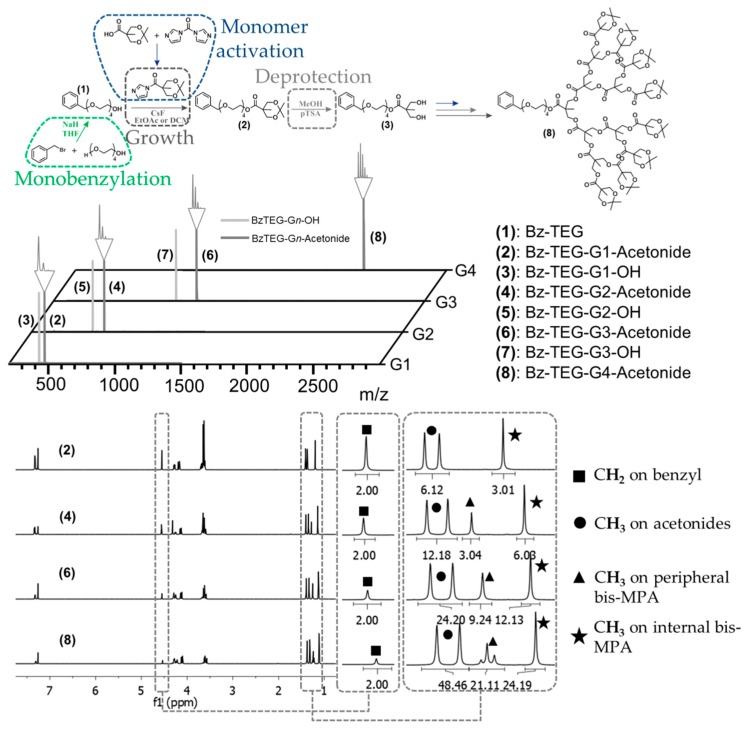
Schematic of the growth of the dendritic scaffold with FPE chemistry with acquired MALDI and NMR spectra, showing the systematic increase in protons per generation.

In this context, tetraethylene glycol (TEG), a short oligoethylene glycol available in large quantity with high purity, was sought out as a building block for the introduction of a primary alcohol at the focal point. Initially, the TEG was monobenzylated by substitution with benzyl bromide in THF using sodium hydride as activation reagent (Figure 1). The monobenzylated tetraethylene glycol (Bz-TEG, **1**) was separated from the dibenzylated byproduct through simple column chromatography giving the pure product in >40 g scale and with a solid yield of 53%. In contrast to earlier proposed divergent growth approaches to bis-MPA dendrons, the successful use of FPE chemistry as growing chemistry is to our knowledge reported herein for the first time ever.

As a result, a divergent growth approach up to the fourth generation dendron Bz-TEG-G4-Acetonide (**8**) with a molecular weight of 2347 Da was successfully executed in seven iterative growth/activation steps and with a total yield of 50%. All growth and deprotection reactions were monitored using MALDI-TOF MS and upon completion the dendrons were isolated using extraction and/or column chromatography purification techniques. The obtained dendrons were isolated in multi gram scale and were properly characterized by ^1^H-, ^13^C-NMR and MALDI techniques (Figure 1).

With the complete set of dendrons accomplished, the third generation dendron **6** was chosen as a model scaffold for orthogonal deprotection and postmodification thanks to the variation of protecting groups at the focal point and periphery. These orthogonal protecting groups were initially evaluated by selective deprotection of the single benzyl group at the focal point. This was accomplished utilizing catalytic hydrogenesis in the presence of palladium catalyst on activated carbon without loss of any peripheral acetonide protection groups. After optimization of the reaction conditions, OH-TEG-G3-Acetonide (**9**) was isolated in an excellent yield of 94%. The facile activation of the TEG-hydroxyl group at the focal point is indeed of importance as it is envisioned to: (i) provide a spacious distance from the dendritic framework, especially for the introduction of bulky groups; (ii) deliver the partial hydrophilicity necessary for biological applications and (iii) allow the functionalization with an array of commercially available molecules with carboxylic acid groups.

To showcase the feasibility of introducing commercially available groups at the focal point, biotin was chosen as a model compound. Biotin is an intriguing functional group due to its high affinity towards the proteins avidin and streptavidin, which gives biotinylated materials relevance for a variety of biomedical applications [27] such as biosensors [28] and tumor localization and cancer therapy [29]. Since both proteins bind a maximum of four biotins, a biotinylated dendron that is peripherally modifiable increases the amount of functionalities that can be adhered to streptavidin. In the case of the third generation dendron, the number of e.g., dyes that could be attached to the protein would increase from 4 to 32, greatly amplifying the effect and aiding detection. A similar concept has been reported utilizing ^18^F-modified PAMAM dendrons with biotinylated focal points. The results revealed that steric hindrance of higher generation dendrons coupled with the constrained biotin core substantially decreased the level of interaction between biotin and avidin [30].

The robust FPE protocol proven successful in the growth was also employed to introduce biotin at the focal point of the bis-MPA dendron. Imidazolide-activated biotin was efficiently reacted to OH-TEG-G3-Acetonide (**9**) and the product Biotin-TEG-G3-Acetonide (**10**) was straightforwardly isolated in 80% yield and through simple extractions. After deprotection of the peripheral acetonide groups under acidic conditions clickable azide groups were further introduced via FPE chemistry with a yield of 78%. The choice of azide groups as peripheral reactive moieties enables reliable postfunctionalization through the highly effective click reactions including CuAAC and strain-promoted alkyne-azide cycloadditions (SPAAC). In this case, disperse red 13 (DR13) was introduced through CuAAC as a model dye. The click reaction was performed in a THF/water mixture and resulted in Biotin-TEG-G3-DR13 (**13**) with a yield of 77%. Dendron **13** was produced from **6** in a total yield of 37% in five reactions with an average yield of 82% per reaction, requiring only one single column chromatography in the final step for purification.

**Figure 2:**
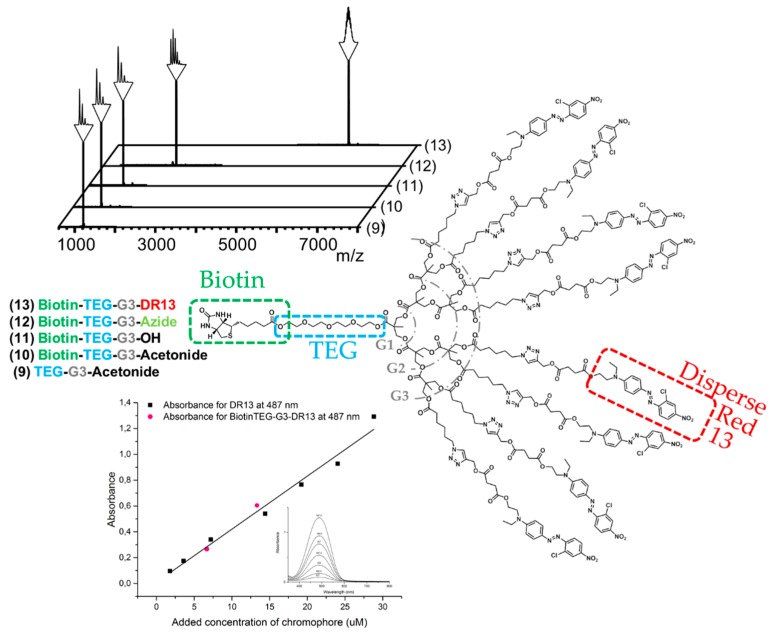
MALDI spectra of the materials obtained through modification of the dendritic scaffold, giving the final biotinylated generation three dendron with eight disperse red dyes at the periphery, and absorbance of acetylene modified disperse red 13 (DR13) and Biotin-TEG-G3-DR13 (13) plotted against concentration. The small insertion shows absorbance spectra of DR13 from 300 to 800 nm.

MALDI spectra acquired throughout the syntheses leading up to the final dendron 13 functionalized with dyes and biotin having a molecular weight of 6242 Da are shown in Figure 2. UV/Vis measurements were performed on the final product to confirm the presence of DR13 and to compare its absorbance to free dye. The measurements confirmed the high purity of the dendron since its measured absorbance at 487 nm (the observed maximum for the acetylene functional DR13) was in line with the standard curve obtained from the free dye, and that the absorbance of an avidin-dendron complex would in fact increase eight-fold in comparison to a complex with simple biotinylated dyes.

## 3. Experimental Section

### 3.1. Materials

All chemicals were purchased from Sigma Aldrich (St. Louis, MO, USA), Merck (Hohenbrunn, Germany), Carbosynth (Compton, UK) VWR (Fontenay-sous-Bois, France) and used as received unless otherwise noted. 6-Azidohexanoic acid and disperse red 13-prop-2-ynyl succinate (acetylene functional disperse red 13) were synthesized according to published procedure [31]. Bis-MPA was kindly donated by Perstorp AB (Perstorp, Sweden).

### 3.2. Nomenclature

Dendrons are in this paper are abbreviated as “Focal functionality”-”Spacer”-“Generation”-“Peripheral functionality”. For example, a third generation dendron with a benzylidene protected TEG spacer and an acetonide protected periphery is denoted as Bz-TEG-G3-Acetonide, and the same generation Dendron, but biotinylated at the focal point with hydroxyl functions in the periphery is denoted Biotin-TEG-G3-OH.

### 3.3. MALDI-TOF

MALDI-TOF spectra were obtained using an UltraFlex MALDI-TOF with SCOUT-MTP Ion Source (Bruker Daltonics, Bremen, Germany) equipped with a nitrogen laser (337 nm), a gridless ion source and a reflector. The instrument was calibrated using SpheriCal™ calibrants. Samples were prepared by mixing 5 µL of 1 g·L^−1^ analyte in EtOAc or MeOH with 5 µL of a 1 g·L^−1^ counter-ion solution of sodium trifluoroacetate (NaTFA) in tetrahydrofuran (THF) and 20 µL of a matrix solution of 10 g·L^−1^ in THF and applying 1 µL this mixture to a stainless steel sample plate using the dried droplet method. The matrix used depended on the sample polarity and was either 9-nitroanthracene (9-NA), *trans*-2-[3-(4-*tert*-butylphenyl)-2-methyl-2-propenylidene]-malononitrile (DCTB) or 2,5-dihydroxybenzoic acid (DHB). The obtained spectra were analyzed with FlexAnaysis version 2.2 from Bruker Daltonics.

### 3.4. ^1^H- and ^13^C-NMR

^1^H-NMR (400 MHz) and ^13^C-NMR (101 MHz) were acquired using a Bruker Avance instrument (Bruker Biospin, Rheinstetten, Germany). ^1^H-NMR spectra were acquired using a spectral window of 20 ppm, a relaxation delay of 1 second and 16 scans. ^13^C-NMR spectra were acquired using a spectral window of 240 ppm, a relaxation delay of 2 seconds and 512 scans. The spectra were processed and analyzed with MestreNova version 9.0.0-12821 from Mestrelab Research (Santiago de Compostela, Spain).

### 3.5. UV/Vis Measurements

DR13 was dissolved in chloroform at the following concentrations (in μM): 1.8, 3.6, 7.2, 14.4, 19.2, 24.0 and 28.8. These solutions were analyzed three times each from 1000 to 300 nm in a UV-2550 UV-vis spectrophotometer (Shimadzu, Kyoto, Japan) and the data was processed with Origin 9.1 (OriginLab, Northampton, MA, USA). The average maxima of the obtained spectra were observed at 487 nm and the absorbance at this wavelength was used to generate a standard curve. Biotin-TEG-G3-DR13 was dissolved in chloroform and analyzed the same way at concentrations of 0.8 and 1.7 μM, which multiplied by the number of chromophores on the dendron (eight) puts it within the values of the standard line.

### 3.6. Cleaning Procedure for Amberlyst A21

The basic resin Amberlyst A21 purchased from Sigma Aldrich (St. Louis, MO, USA) was cleaned prior to being used. This was performed through suspending it twice in solvent for 1 h through stirring with a magnet, and then a third time for 14 h. This was performed with DCM, EtOAc and methanol in that order. Between washings the solvent was removed by filtration through a pore 4 solid filter.

### 3.7. Syntheses

*Acetonide protected bis-MPA (bis-MPA-Acetonide).* Bis-MPA (300 g, 2.24 mol, 1 eq.) was dissolved in acetone (2.4 L). 2,2-Dimethylolpropane (2,2-DMP, 416 mL, 3.36 mol, 1.5 eq.) and *p*-toluenesulfonic acid (*p*TSA) monohydrate (2.55 g, 13.4 mmol, 0.006 eq.) were added in that order. The reaction was carried out at room temperature for 14 h and was then quenched by adding a solution of NH_3_/EtOH (1:1, 6 mL). The acetone was evaporated and the product was dissolved in DCM. The organic phase was washed five times with deionized water, dried over MgSO_4_, filtered and the solvent was evaporated giving bis-MPA-Acetonide (205 g, 1.18 mol, 52.7%) as a white solid. ^1^H-NMR: (CDCl_3_) δ 4.19 (d, 2H, C-C**H_2_**-O), 3.67 (d, 2H, C-C**H_2_**-O), 1.42 (d, 6H, C-C**H_3_**), 1.21 (s, 3H, C-C**H_3_**). ^13^C-NMR: (101 MHz, CDCl_3_) δ 180.50, 98.44, 65.94, 41.88, 25.21, 22.19, 18.58.

*Monobenzylated tetraethylene glycol* (Bz-TEG, **1**). In a two necked round-bottomed flask, TEG (97.0 g, 500 mol, 1.67 eq.) was dissolved in THF (325 mL) and immersed in an ice-bath. Sodium hydride (60% in mineral oil dispersion, 20.8 g, 520 mmol, 1.04 eq.) was added slowly. The mixture was stirred for 30 min at room temperature, after which benzyl bromide (51.2 g, 300 mmol, 1 eq.) was added dropwise. The reaction was carried out for 14 h at room temperature and then quenched with H_2_O (145 mL). The product was extracted with 4 × 70 mL of EtOAc and the combined organic phases were dried with MgSO_4_ and rotoevaporated. The product was purified by silica gel chromatography using 1:1 EtOAc–heptane (R*_f_* 0.14) as eluent, which gave pure monobenzylated tetraethylene glycol (45 g, 158 mmol, 52.7%) as a yellow oil. ^1^H-NMR: (CDCl_3_): δ 7.39–7.23 (m, 5H, C_6_**H_5_**-CH_2_), 4.57 (s, 2H, C_6_**H**_5_-C**H_2_**-O), 3.78–3.56 (m, 16H, C**H_2_**-C**H_2_**-O), 2.62 (s, 1H, CH_2_-O**H**). ^13^C-NMR: (CDCl_3_) δ 138.10, 128.19, 127.58, 127.43, 73.04, 72.43, 70.47, 70.45, 70.41, 70.17, 69.28, 61.46.

General procedure for dendritic growth. In a round bottom flask, 1.3–1.5 equivalents of acetonide protected bis-MPA per hydroxyl group on the dendron were suspended in DCM to a concentration of 1 M. The same amount of equivalents of CDI was added, and the reaction was carried out for 1 h after which the formation of imidazolide-activated acetonide protected bis-MPA was confirmed by ^1^H-NMR (CDCl_3_): δ 8.28 (s, 1H, N-C**H**=N), 7.55 (t, *J* = 1.5 Hz, 1H, N-C**H**=CH), 7.09 (dd, *J* = 1.7, 0.8 Hz, 1H, CH=C**H**-N)). Next one equivalent of hydroxyl functional dendron and a catalytic amount of CsF were added to the flask and the reaction was carried out for 14 h. Complete conversion was confirmed with MALDI using DCTB or 9-NA as matrix and Cs^+^ as counterion (already present in the mixture). The reaction was quenched through addition of water, and after confirmation of the quenching with ^1^H-NMR the organic phase was diluted and washed 4 times with 10% aqueous NaHSO_4_ and 10% aqueous Na_2_CO_3_, dried with MgSO_4_, filtered and the solvent was rotoevaporated. Column chromatography was performed when specifically stated.

General procedure for acetonide deprotection. The acetonide protected dendron was added to a round bottom flask and dissolved in a large excess of methanol. 0.5 wt % of *p*TSA monohydrate was added and the solution was carefully rotoevaporated to remove the acetone formed. When complete conversion was confirmed with MALDI, the solution was filtered through a column of Amberlyst A21 to remove the pTSA, after which the solvent was evaporated.

*Bz-TEG-G1-Acetonide* (**2**). Bis-MPA-Acetonide (40.5 g, 232 mmol, 1.5 eq.), CDI (37.7 g, 232 mmol, 1.5 eq.), monobenzylated tetraethylene glycol (44.0 g, 155 mmol, 1 eq.) and CsF (1.20 g, 7.90 mmol, 0.05 eq.) were reacted according to the general procedure for dendritic growth. Bz-TEG-G1-Acetonide (62.0 g, 141 mmol, 91.0%) was obtained as a yellow oil. ^1^H-NMR: (CDCl_3_) δ 7.37–7.21 (m, 5H, C_6_**H_5_**-CH_2_), 4.55 (s, 2H C_6_**H**_5_-C**H_2_**-O), 4.32–4.24 (m, 2H, CH_2_-C**H_2_**-O), 4.18 (d, *J* = 11.8 Hz, 2H, C-C**H_2_**-O), 3.83–3.58 (m, 16H, C**H_2_**-C**H_2_**-O, C-C**H_2_**-O), 1.44–1.33 (m, 6H, C-C**H_3_**), 1.20 (s, 3H, C-C**H_3_**). ^13^C-NMR: (CDCl_3_) δ 174.08, 138.25, 128.31, 127.68, 127.54, 97.98, 73.18, 70.62, 70.60, 70.57, 69.41, 68.97, 65.90, 63.85, 41.75, 24.22, 23.07, 18.66. MALDI: *m*/*z* calc. 440.24 Da. Found [M + Na]^+^ 464.37 Da.

*Bz-TEG-G1-OH* (**3**). Bz-TEG-G1-Acetonide (60.9 g, 138 mmol, 1 eq.) and *p*TSA monohydrate (525 mg, 2.76 mmol, 0.02 eq.) were reacted according to general procedure for acetonide deprotection. The product was purified with silica gel chromatography using 1:1 EtOAc–heptane (R*_f_* 0.1) as eluent, which gave pure Bz-TEG-G1-OH (48.4 g, 121 mmol, 87.7%) as a yellow oil. ^1^H-NMR: (CDCl_3_) δ 7.37–7.22 (m, 5H, C_6_**H_5_**-CH_2_), 4.56 (s, 2H, C_6_**H**_5_-C**H_2_**-O), 4.37–4.30 (m, 2H, CH_2_-C**H_2_**-O), 3.87–3.59 (m, 18H, C**H**_2_-C**H_2_**-O, C-C**H_2_**-O), 2.98 (t, *J* = 6.7 Hz, 2H, CH_2_-O**H**), 1.10 (s, 3H, C-C**H_3_**). ^13^C-NMR: (CDCl_3_) δ 175.37, 138.02, 128.18, 127.57, 127.44, 77.48, 77.16, 76.84, 73.01, 70.40, 70.37, 70.34, 70.21, 69.22, 68.60, 66.52, 63.21, 49.56, 16.97. MALDI: *m*/*z* calc. 400.21 Da. Found [M + Na]^+^ 423.53 Da.

*Bz-TEG-G2-Acetonide* (**4**). Bis-MPA-Acetonide (49.8 g, 286 mmol, 2.6 eq.), CDI (46.3 g, 286 mmol, 2.6 eq.), Bz-TEG-G1-OH (44.0 g, 110 mmol, 1 eq.) and CsF (3.40 g, 22.4 mmol, 0.2 eq.) were reacted according to the general procedure for dendritic growth. Bz-TEG-G2-Acetonide (76.2 g, 107 mmol, 97.2%) was obtained as a yellow oil. ^1^H-NMR: (CDCl_3_δ 7.37–7.25 (m, 5H, C_6_**H_5_**-CH_2_), 4.56 (s, 2H, C_6_**H**_5_-C**H_2_**-O), 4.32 (s, 4H, C-C**H_2_**-O), 4.29–4.24 (t, 2H, CH_2_-C**H_2_**-O), 4.19–4.11 (d, 4H, C-**CH_2_**-O), 3.75–3.57 (m, 18H, C**H_2_**-C**H_2_**-O, C-C**H_2_**-O), 1.38 (d, *J* = 22.4 Hz, 12H, C-C**H_3_**), 1.29 (s, 3H, C-C**H_3_**), 1.15 (s, 6H, C-C**H_3_**). ^13^C-NMR: (101 MHz, CDCl_3_) ^13^C-NMR (CDCl_3_) δ 173.54, 172.55, 138.33, 128.39, 127.77, 127.63, 98.13, 73.27, 70.70, 70.65, 70.63, 69.49, 68.88, 66.02, 65.98, 65.30, z64.32, 46.78, 42.08, 25.05, 22.32, 18.60, 17.77. MALDI: *m*/*z* calc 723.37 Da. Found [M + Na]^+^: 737.36 Da.

*Bz-TEG-G2-OH* (**5**). Bz-TEG-G2-Acetonide (65.9 g, 92.4 mmol, 1 eq.) and *p*TSA monohydrate (351 mg, 1.85 mmol, 0.02 eq.) were reacted according to general procedure for acetonide deprotection. Bz-TEG-G2-OH (52.5, 83.0 mmol, 89.8%) was obtained as a yellow oil. ^1^H-NMR: (CDCl_3_) δ 7.39–7.29 (m, 5H, C_6_**H_5_**-CH_2_), 4.58 (s, 2H, C_6_**H**_5_-C**H_2_**-O), 4.46–4.27 (m, 6H, C-C**H_2_**-O, CH_2_-C**H_2_**-O), 3.82 (m, 4H, C-C**H_2_**-OH), 3.77–3.61 (m, 18H, C**H_2_**-C**H_2_**-O, C-C**H_2_**-OH), 3.24 (dt, *J* = 18.7, 6.2 Hz, 4H, CH_2_-O**H**), 1.33 (s, 3H, C-C**H_3_**), 1.08 (s, 6H, C-C**H_3_**). ^13^C-NMR: (CDCl_3_) δ 173.54, 172.55, 138.33, 128.39, 127.77, 127.63, 98.13, 73.27, 70.70, 70.65, 70.63, 69.49, 68.88, 66.02, 65.98, 65.30, 64.32, 46.78, 42.08, 25.05, 22.32, 18.60, 17.77. MALDI: *m*/*z* calc 632.30 Da. Found [M + Na]^+^ 656.26 Da.

*Bz-TEG-G3-Acetonide* (**6**). Bis-MPA-Acetonide (63.6 g, 365 mmol, 5.2 eq.), CDI (59.2 g, 365 mmol, 5.2 eq.), Bz-TEG-G2-OH (44.4 g, 70.2 mmol, 1 eq.) and CsF (3.13 g, 20.6 mmol, 0.3 eq.) were reacted according to the general procedure for dendritic growth. The product was purified with silica gel chromatography using 2:3 EtOAc–heptane as eluent (R*_f_* 0.4 in 4:1 EtOAc–heptane), which gave pure Bz-TEG-G3-Acetonide (74.8 g, 59.5 mmol, 84.8%) was obtained as a clear oil. ^1^H-NMR: (CDCl_3_) δ 7.35–7.27 (m, 5H, C_6_**H_5_**-CH_2_), 4.55 (s, 2H, C_6_**H**_5_-C**H_2_**-O), 4.34–4.21 (m, 14H, C-C**H_2_**-O, CH_2_-C**H_2_**-O), 4.13 (d, *J* = 11.8 Hz, 8H, C-C**H_2_**-O), 3.71–3.56 (m, 22H, C-C**H_2_**-O, C**H_2_**-C**H_2_**-O), 1.37 (d, *J* = 24.0 Hz, 24H, C-C**H_3_**), 1.26 (s, 9H, C-C**H_3_**), 1.13 (s, 12H, C-C**H_3_**). ^13^C-NMR: (CDCl_3_) δ 173.58, 172.15, 171.93, 138.36, 128.45, 127.83, 127.69, 98.19, 73.33, 70.75, 70.72, 70.69, 70.57, 69.52, 68.87, 66.07, 66.02, 65.97, 64.99, 64.49, 46.94, 46.69, 42.13, 25.28, 22.18, 18.61, 17.79, 17.70. MALDI: *m*/*z* calc. 1256.62 Da. Found [M + Na]^+^ 1279.88 Da.

*Bz-TEG-G3-OH* (**7**). Bz-TEG-G3-Acetonide (8.29 g, 6.59 mmol, 1 eq.) and *p*TSA monohydrate (25.0 mg, 130 μmol, 0.02 eq.) were reacted according to general procedure for acetonide deprotection. Bz-TEG-G3-OH (6.72 g, 6.12 mmol, 92.9%) was obtained as a white solid. ^1^H-NMR: (CD_3_OD) δ 7.43–7.21 (m, 5H, C_6_**H_5_**-CH_2_), 4.56 (s, 2H, C_6_**H**_5_-C**H_2_**-O), 4.40–4.18 (m, 13H, CH_2_-C**H_2_**-O, C-C**H_2_**-O), 3.77–3.55 (m, 29H, C-C**H_2_**-O, C**H_2_**-C**H_2_**-O), 1.30 (s, 3H, C-C**H_3_**), 1.29 (s, 6H, C-C**H_3_**), 1.15 (s, 14H, C-C**H_3_**). ^13^C-NMR: (CD_3_OD) δ 175.83, 173.86, 173.68, 139.58, 129.35, 128.88, 128.66, 79.43, 74.09, 71.54, 71.52, 71.45, 70.58, 69.83, 67.20, 66.11, 65.78, 65.63, 51.73, 47.87, 47.85, 18.24, 18.07, 17.30. MALDI: *m*/*z* calc 1096.49 Da. Found [M + Na]^+^ 1117.87 Da.

*Bz-TEG-G4-Acetonide* (**8**). Bis-MPA-Acetonide (3.05 g, 17.5 mmol, 12 eq.), CDI (2.84 g, 17.5 mmol, 12 eq.), Bz-TEG-G3-OH (1.60 g, 1.46 mmol, 1 eq.) and CsF (354 mg, 2.30 mmol, 1.6 eq.) were reacted according to the general procedure for dendritic growth Bz-TEG-G4-Acetonide (3.11 g, 1.34 mmol, 91.8%) was obtained as a clear viscous solid. ^1^H-NMR (CDCl_3_) δ 7.35–7.20 (m, 5H, C_6_**H_5_**-CH_2_), 4.53 (s, 2H, C_6_**H**_5_-C**H_2_**-O), 4.34–4.16 (m, 30H, CH_2_-C**H_2_**-O, C-C**H_2_**-O), 4.11 (d, *J* = 11.8 Hz, 16H, C-C**H_2_**-O), 3.70–3.54 (m, 30H, C-C**H_2_**-O, C**H_2_**-C**H_2_**-O), 1.35 (d, *J* = 24.5 Hz, 48H, C-C**H_3_**), 1.26 (s, 3H, C-C**H_3_**), 1.24 (s, 12H, C-C**H_3_**), 1.22 (s, 6H, C-C**H_3_**), 1.11 (s, 24H, C-C**H_3_**). ^13^C-NMR (CDCl_3_) δ 173.51, 171.92, 171.86, 171.44, 138.34, 128.41, 127.78, 127.65, 98.14, 73.28, 70.71, 70.68, 70.65, 70.51, 69.50, 68.81, 66.31, 66.03, 65.97, 65.56, 64.87, 64.48, 46.89, 46.76, 46.68, 42.09, 25.22, 22.19, 18.59, 17.76, 17.58, 17.54. MALDI: *m*/*z* calc.: 2346.61 Da. Found [M + Na]^+^: 2368.67 Da.

*OH-TEG-G3-Acetonide* (**9**). Palladium on carbon (10 wt % loaded, 15.0 mg, 5 wt %) was suspended in EtOAc (20 mL) and added to a round bottom flask together with Bz-TEG-G3-Acetonide (304 mg, 242 μmol, 1 eq.). The flask was evacuated and hydrogen gas was introduced. The reaction was carried out for 20 minutes under heavy stirring. The palladium on carbon was filtered off and the solvent was evaporated, giving OH-TEG-G3-Acetonide (266 mg, 228 μmol, 94.2%) as a clear oil. ^1^H-NMR: (CDCl_3_) δ 4.27–4.12 (m, 14H, CH_2_-C**H_2_**-O, C-C**H_2_**-O), 4.03 (d, *J* = 11.8 Hz, 8H, C-C**H_2_**-O), 3.65–3.43 (m, 23H, C-C**H_2_**-O, C**H_2_**-C**H_2_**-O), 1.27 (d, *J* = 25.3 Hz, 24H, C-C**H_3_**), 1.17 (d, *J* = 1.8 Hz, 6H, C-C**H_3_**), 1.03 (s, 12H, C-C**H_3_**). ^13^C-NMR: (CDCl_3_) δ 173.34, 171.95, 171.71, 170.90, 97.93, 72.42, 70.48, 70.41, 70.34, 70.21, 68.66, 65.82, 65.77, 64.77, 64.27, 61.49, 60.20, 46.73, 46.47, 41.90, 25.12, 21.88, 20.88, 18.35, 17.55, 17.47, 14.08. MALDI: *m*/*z* calc.: 1166.57 Da. Found [M + Na]^+^ 1188.91 Da.

*Biotin-TEG-G3-Acetonide* (**10**). Biotin (98.0 mg, 200 µmol, 2 eq.) was suspended in a solution of 1:1 EtOAc–DMF (100 μL) and heated to 45 °C. CDI (650 mg, 200 µmol, 2 eq.) was added and the formation of imidazolide-activated biotin was carried out for 1 h after which OH-TEG-G3-Acetonide (234 mg, 200 µmol, 1 eq.) and CsF (6.00 mg, 40.0 µmol, 0.2 eq.) was added. The reaction was carried out for 14 h and after confirmation of the conversion with MALDI, the reaction was quenched with water and the organic phase was diluted and washed four times with 10% aqueous NaHSO_4_ and 10% aqueous Na_2_CO_3_, dried with MgSO_4_, filtered and the solvent was rotoevaporated, which gave Biotin-TEG-G3-Ac (219 mg, 160 µmol, 80.0%) as a clear solid. ^1^H-NMR: (CDCl_3_) δ 5.81 (s, 1H, CH-N**H**-CO), 5.35 (s, 1H, CH-N**H**-CO) 4.48 (dd, *J* = 7.8, 5.0 Hz, 1H, NH-C**H**-CH_2_), 4.35–4.17 (m, 15H, CH_2_-C**H_2_**-O, C-C**H_2_**-O, NH-C**H**-CH), 4.13 (d, *J* = 11.8 Hz, 8H, C-C**H_2_**-O), 3.65–3.56 (m, 18H, C-C**H_2_**-O, C**H_2_**-C**H_2_**-O), 3.15 (ddd, *J* = 8.5, 6.4, 4.5 Hz, 1H, ((S-, CH-)C**H**-CH_2_), 2.90 (dd, *J* = 12.8, 5.0 Hz, 1H, CH-C**H_2_**-S), 2.73 (d, *J* = 12.8 Hz, 1H, CH-C**H_2_**-S), 2.35 (t, *J* = 7.5 Hz, 2H, CH_2_-C**H_2_**-O), 1.90–1.42 (m, 6H, C**H_2_**-C**H_2_**-C**H_2_**), 1.37 (d, *J* = 24.8 Hz, 23H, C-C**H_3_**), 1.26 (s, 9H, C-C**H_3_**), 1.13 (s, 12H, C-C**H_3_**). ^13^C-NMR: ^13^C-NMR (CDCl_3_) δ 173.55, 173.48, 172.04, 171.83, 163.23, 162.52, 98.09, 77.36, 77.25, 77.04, 76.73, 70.58, 70.48, 70.46, 69.14, 68.77, 65.97, 65.91, 64.89, 64.36, 63.41, 61.88, 60.08, 55.37, 46.84, 46.59, 42.04, 40.53, 36.48, 33.74, 31.43, 28.29, 28.24, 25.20, 24.70, 22.05, 18.51, 17.68, 17.60. MALDI: *m*/*z* calc.: 1392.65 Da. Found [M + Na]^+^: 1414.31 Da.

*Biotin-TEG-G3-OH* (**11**). Biotin-TEG-G3-Acetonide (200 mg, 144 µmol, 1 eq.) was dissolved in an excess of methanol. *p*TSA monohydrate (15.0 mg, 79.0 µmol, 0.55 eq.) was added and the mixture was rotoevaporated carefully. When conversion was confirmed with MALDI, the reaction mixture was filtered through a column of Amberlyst A21, the solvent was evaporated giving Biotin-TEG-G3-OH (145 mg, 118 µmol, 81.9%) as a clear solid. ^1^H-NMR: (CD_3_OD) δ 4.51 (dd, *J* = 8.0, 4.9 Hz, 1H, NH-C**H**-CH_2_), 4.42–4.17 (m, 17H, CH_2_-C**H_2_**-O, C-C**H_2_**-O, NH-C**H**-CH), 3.81–3.54 (m, 28H, C-C**H_2_**-OH, C**H_2_**-C**H_2_**-O), 3.27–3.18 (m, 1H, ((S-, CH-)C**H**-CH_2_), 2.94 (dd, *J* = 12.8, 4.9 Hz, 1H, CH-C**H_2_**-S), 2.72 (d, *J* = 12.7 Hz, 1H, CH-C**H_2_**-S), 2.38 (t, *J* = 7.3 Hz, 2H, CH_2_-C**H_2_**-O), 1.83–1.37 (m, 6H, C**H_2_**-C**H_2_**-C**H_2_**), 1.32 (s, 3H, C-C**H_3_**), 1.30 (s, 6H, C-C**H_3_**), 1.15 (s, 12H, C-C**H_3_**). ^13^C-NMR: (CD_3_OD) δ 175.84, 175.22, 173.87, 173.69, 165.97, 71.54, 71.51, 71.47, 71.45, 70.09, 69.84, 67.21, 66.12, 65.76, 65.64, 64.59, 63.32, 61.56, 56.90, 51.72, 47.87, 47.85, 41.03, 34.72, 34.69, 29.63, 29.42, 25.87, 25.80, 18.25, 18.10, 17.32. MALDI: *m*/*z* calc.: 1232.52 Da. Found [M + Na]^+^: 1254.24 Da.

*Biotin-TEG-G3-Azide* (**12**). 6-Azidohexanoic acid (245 mg, 1.56 mmol, 13.7 eq.) was dissolved in DCM in a vial. CDI (253 mg, 1.56 mmol, 13.7 eq.) was added and the formation of imidazole-activatated 6-azidohexanoic acid was carried out for 1 h after which Biotin-TEG-G3-OH (140 mg, 113 µmol. 1 eq.) and CsF (28.0 mg, 182 µmol, 1.6 eq.) were added. The reaction was carried out for 16 h and after confirmation of the conversion with MALDI, the reaction was quenched with water and the organic phase was diluted and washed eight times with 10% aqueous NaHSO_4_ and eight times with 10% aqueous Na_2_CO_3_, dried with MgSO_4_, filtered and the solvent was rotoevaporated, which gave Biotin-TEG-G3-Azide (206 mg, 88.0 µmol, 77.9%) as a yellow oil. ^1^H-NMR: (CDCl_3_) δ 5.70 (s, 1H, CH-N**H**-CO), 5.33–5.21 (m, 1H, CH-N**H**-CO), 4.46 (dd, *J* = 7.8, 5.1 Hz, 1H, NH-C**H**-CH_2_), 4.35–4.03 (m, 32H, C**H_2_**-C**H_2_**-O, C-C**H_2_**-O), 3.72–3.54 (m, 13H, C**H_2_**-C**H_2_**-O), 3.23 (t, *J* = 6.8 Hz, 18H, C**H_2_**-C**H_2_**-N_3_), 3.12 (ddd, *J* = 8.4, 6.4, 4.5 Hz, 1H, ((S-, CH-)C**H**-CH_2_), 2.87 (dd, *J* = 12.8, 4.8 Hz, 1H, CH-C**H_2_**-S), 2.70 (d, *J* = 12.8 Hz, 1H, CH-C**H_2_**-S), 2.35–2.24 (m, 19H, CH_2_-C**H_2_**-CO), 1.79–1.27 (m, 58H, C**H_2_**-C**H_2_**-C**H_2_**), 1.25 (s, 3H, C-C**H_3_**), 1.20 (s, 6H, C-C**H_3_**), 1.18 (s, 12H, C-C**H_3_**). ^13^C-NMR: (CDCl_3_) δ 173.53, 172.75, 171.97, 171.91, 171.44, 163.40, 70.57, 70.47, 70.44, 69.15, 68.76, 66.09, 65.17, 64.92, 64.38, 63.37, 61.90, 60.11, 55.45, 51.53, 51.22, 51.18, 46.68, 46.56, 46.38, 40.53, 33.83, 33.74, 29.67, 28.53, 28.32, 28.25, 26.24, 26.18, 24.73, 24.43, 24.31, 17.77, 17.53, 17.48. MALDI: *m*/*z* calc.: 2346.60 Da. Found [M + Na]^+^: 2372.88 Da.

*Biotin-TEG-G3-DR13* (**13**). Biotin-TEG-G3-Azide (25.0 mg, 10.7 μmol, 1 eq.) and acetylene functional disperse red 13 (50 mg, 102.3 μmol, 9.6 eq.) were dissolved in THF (100 μL). Sodium ascorbate (3.38 mg, 17.1 μmol, 1.6 eq.) and copper sulfate (1.36 mg, 8.50 μmol, 0.8 eq.) were dissolved in H_2_O (100 μL) and added to the reaction mixture. The reaction was carried out at 50 °C for 1 h. The product was extracted with 4 × 10 mL of chloroform. The combined organic phases were dried with MgSO_4_ and the solvent was evaporated. The product was purified with gradient silica gel chromatography (R*_f_* 0.85 in 1:9 MeOH–DCM) from 1:9 EtOAc–heptane Δ10% to pure EtOAc followed by 1:9 MeOH-DCM. Biotin-TEG-G3-DR13 was obtained as a red solid (51.0 mg, 8.17 μmol, 76.4%) ^1^H-NMR: (CDCl_3_) δ 8.34 (d, *J* = 2.4 Hz, 8H, C(Cl)=C**H-**C(NO_2_)), 8.11 (dd, *J* = 8.9, 2.5 Hz, 8H, CH-C**H**=C(NO_2_)), 7.97–7.85 (m, 16H, (CH-C**H**=C(N)), 7.74 (d, *J* = 9.0 Hz, 8H, C(N)=C**H**-CH), 7.61 (s, 8H, N-C**H**=C), 6.84–6.70 (m, 16H, CH-C**H**=C(N)), 5.20 (s, 16H, C-C**H_2_**-O), 4.48 (t, *J* = 6.5 Hz, 1H, NH-C**H**-CH_2_), 4.44–4.04 (m, 65H, CH_2_-C**H_2_**-O, C-C**H_2_**-O, CH_2_-C**H_2_**-N), 3.72–3.56 (m, 29H, C**H_2_**-C**H_2_**-O, C**H_2_**-C**H_2_**-N), 3.52 (q, *J* = 7.1 Hz, 16H, CH_3_-C**H_2_**-N), 3.14 (q, *J* = 7.5, 6.9 Hz, 1H, ((S-, CH-)C**H**-CH_2_), 2.89 (dd, *J* = 12.9, 4.9 Hz, 1H, CH-C**H_2_**-S), 2.70 (d, *J* = 12.8 Hz, 1H, CH-C**H_2_**-S) 2.62 (s, 33H, CH-C**H_2_**-S), 2.28 (t, *J* = 7.4 Hz, 18H, CH_2_-C**H_2_**-CO), 1.87 (q, *J* = 7.5 Hz, 16H, CH_2_-C**H_2_**-CH_2_), 1.60 (p, *J* = 7.5 Hz, 19H, CH_2_-C**H_2_**-CH_2_), 1.48–1.14 (m, 70H, C-C**H_3_**, C**H_2_**-C**H_2_**-C**H_2_**, CH_2_-C**H_3_, **C**H_2_**-C**H_2_**-C**H_2_**). MALDI: *m*/*z* calc.: 6241.87 Da. Found [M + Na]^+^: 6264.51 Da.

## 4. Conclusions

Monodisperse dendrons based on bis-MPA have been straightforwardly synthesized up to the fourth generation via a divergent growth approach utilizing FPE chemistry as the key esterification reaction. In contrast to traditional bis-MPA dendrons with a constrained carboxylic acid at the focal point, the synthesized dendrons displayed a primary alcohol being distanced from the framework by a hydrophilic tetraethylene glycol linker. By introducing an alcohol as core group, robust functionalizations can be performed with commercially available compounds bearing carboxylic acid groups and with minimum loss of valuable dendrons. As a proof-of-concept, the third generation dendron was biotinylated in the core as well as peripherally azido functionalized using FPE chemistry with overall high yields and specificity. Finally, bulky disperse red 13 dye was introduced exploiting the highly efficient CuAAC click reaction. The final dendron of 6241.87 Da displayed all elements of an advanced dendritic probe suited for biological interactions with the proteins streptavidin or avidin.

The presented route to versatile high purity dendrons in large scales through FPE chemistry with great potential for biomedical material applications through efficient demonstrated functionalizations is a significant improvement over established protocols since it involves a lower consumption of toxic reagents, more efficient monomer usage and facile purifications.
